# Characterization of the multimeric structure of poly(A)-binding protein on a poly(A) tail

**DOI:** 10.1038/s41598-018-19659-6

**Published:** 2018-01-23

**Authors:** Ryoichi Sawazaki, Shunsuke Imai, Mariko Yokogawa, Nao Hosoda, Shin-ichi Hoshino, Muneyo Mio, Kazuhiro Mio, Ichio Shimada, Masanori Osawa

**Affiliations:** 1 Graduate School of Pharmaceutical Sciences, Keio University, Shibakoen, Minato-ku, Tokyo, 105-8512 Japan; 2 Graduate School of Pharmaceutical Sciences, The University of Tokyo, Hongo, Bunkyo-ku, Tokyo, 113-0033 Japan; 3 Graduate School of Pharmaceutical Sciences, Nagoya City University, Tanabe-dori, Mizuho-ku, Nagoya, 467-8603 Japan; 4 Molecular Profiling Research Center for Drug Discovery and OPERANDO Open Innovation Laboratory, National Institute of Advanced Industrial Science and Technology, Koto-ku, Tokyo, 135-0064 Japan

## Abstract

Eukaryotic mature mRNAs possess a poly adenylate tail (poly(A)), to which multiple molecules of poly(A)-binding protein C1 (PABPC1) bind. PABPC1 regulates translation and mRNA metabolism by binding to regulatory proteins. To understand functional mechanism of the regulatory proteins, it is necessary to reveal how multiple molecules of PABPC1 exist on poly(A). Here, we characterize the structure of the multiple molecules of PABPC1 on poly(A), by using transmission electron microscopy (TEM), chemical cross-linking, and NMR spectroscopy. The TEM images and chemical cross-linking results indicate that multiple PABPC1 molecules form a wormlike structure in the PABPC1-poly(A) complex, in which the PABPC1 molecules are linearly arrayed. NMR and cross-linking analyses indicate that PABPC1 forms a multimer by binding to the neighbouring PABPC1 molecules via interactions between the RNA recognition motif (RRM) 2 in one molecule and the middle portion of the linker region of another molecule. A PABPC1 mutant lacking the interaction site in the linker, which possesses an impaired ability to form the multimer, reduced the *in vitro* translation activity, suggesting the importance of PABPC1 multimer formation in the translation process. We therefore propose a model of the PABPC1 multimer that provides clues to comprehensively understand the regulation mechanism of mRNA translation.

## Introduction

In human cells, most messenger RNAs (mRNAs) in the cytoplasm initially possess a 5′ cap and a 3′ poly adenylate (poly(A)) tail with an approximate length of 200 bases^1,2^. The poly(A) is gradually digested from the 3′ end, and it is reported that the poly(A) lengths of 50–100 bases are most populated in the steady state of the Hela and NIH3T3 cells^3,4^. The 3′-poly(A) tail is covered by multiple molecules of cytoplasmic poly(A)-binding protein C1 (PABPC1)^5,6^.

**Figure 1:**
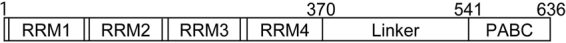
Domain composition of PABPC1.

PABPC1 is a 636-residue basic protein, comprising N-terminal four tandemly repeated RNA recognition motifs (RRMs), each of which consists of approximately 90 residues, followed by a proline-rich unstructured linker region possessing approximately 170 residues, and a C-terminal PABC domain with 75 residues^7–10^ (Fig. 1). A single molecule of PABPC1 reportedly binds to a 23–27-base region of poly(A) via the four RRMs, with a dissociation constant (*K*_d_) of 4 nM^6,11^. The cytoplasmic concentration of PABPC1 is reportedly 10^–6^ M^12^, which is 10^3^-fold of the *K*_d_ value. Therefore, PABPC1 is assumed to cover whole poly(A), where a poly(A) with a length of 200 bases is assumed to accommodate on average 8 molecules of PABPC1^6,11^. The poly(A)-bound PABPC1 molecules are multimerized via mutual intermolecular interactions, which reportedly involves the linker region^9,13^.

Various proteins have been reported to bind to the poly(A)-bound PABPC1: eIF4G in the eIF4F translation initiation complex^14^, eRF3 in the translation termination complex (eRF1-eRF3)^15^, translation enhancer Paip1^16^, translation repressor Paip2^17^, and Tob and Pan3 in the deadenylase complex (Tob-Caf1 and Pan2-Pan3)^18–20^. These proteins play various and important roles in translation and mRNA metabolism^21^.

The structural bases of some of these interactions have been elucidated, such as a ternary complex of the RRM region corresponding to RRM1 and RRM2, poly(A), and eIF4G (residues 179–198)^22^, as well as PABC in complex with peptide from e.g., Paip1^23^, Paip2^23,24^, eRF3^25,26^. These analyses have utilized monomeric PABPC1, without consideration of the multimerized PABPC1 structure. Whereas a number of the regulatory proteins simultaneously associate with multimeric PABPC1 on poly(A), it remains elusive which PABPC1 molecules interact with each of the regulatory proteins and whether the interactions are cooperative, competitive, or independent. Therefore, to elucidate the mechanisms of translational regulation associated with these PABPC1-binding proteins, it is necessary to know the structure of the PABPC1 multimerized on poly(A), which has remained unsolved.

In the present study, we physicochemically characterized the multimerized structure of the poly(A)-bound PABPC1 by using transmission electron microscopy (TEM), chemical cross-linking, and nuclear magnetic resonance (NMR) spectroscopy. The molecular basis of the multimer formation of the poly(A)-bound PABPC1 multimer revealed here contributes to an understanding how multiple and various PABPC1-binding proteins bind to either of the PABPC1 molecules on poly(A), as this is the state in which the subsequent functions are realized.

## Results

### Transmission electron microscopy of (multiple) PABPC1-poly(A) complex

Human PABPC1 was expressed in *E. coli*, and purified to homogeneity. The purified protein exhibited poly(A)-binding affinity with a dissociation constant of 6.9 × 10^−10^ M (Supplementary Figure 1a, and Table 1), which is consistent with a previous report^11^.

**Table 1:** Binding parameters for the interactions of purified full length PABPC1 and RRM1/2/3/4 with poly(A), obtained by surface plasmon resonance analysis.

	*k*_ass_ (1/Ms)	*k*_dis_ (1/s)	*K*_d_ (M)	χ^2^
PABPC1	(1.494 ± 0.005) × 10^6^	(1.032 ± 0.001) × 10^−3^	(6.91 ± 0.03) × 10^−10^	0.29
RRM1/2/3/4	(4.13 ± 0.06) × 10^6^	(6.12 ± 0.05) × 10^−4^	(1.49 ± 0.03) × 10^−10^	0.085

**Figure 2:**
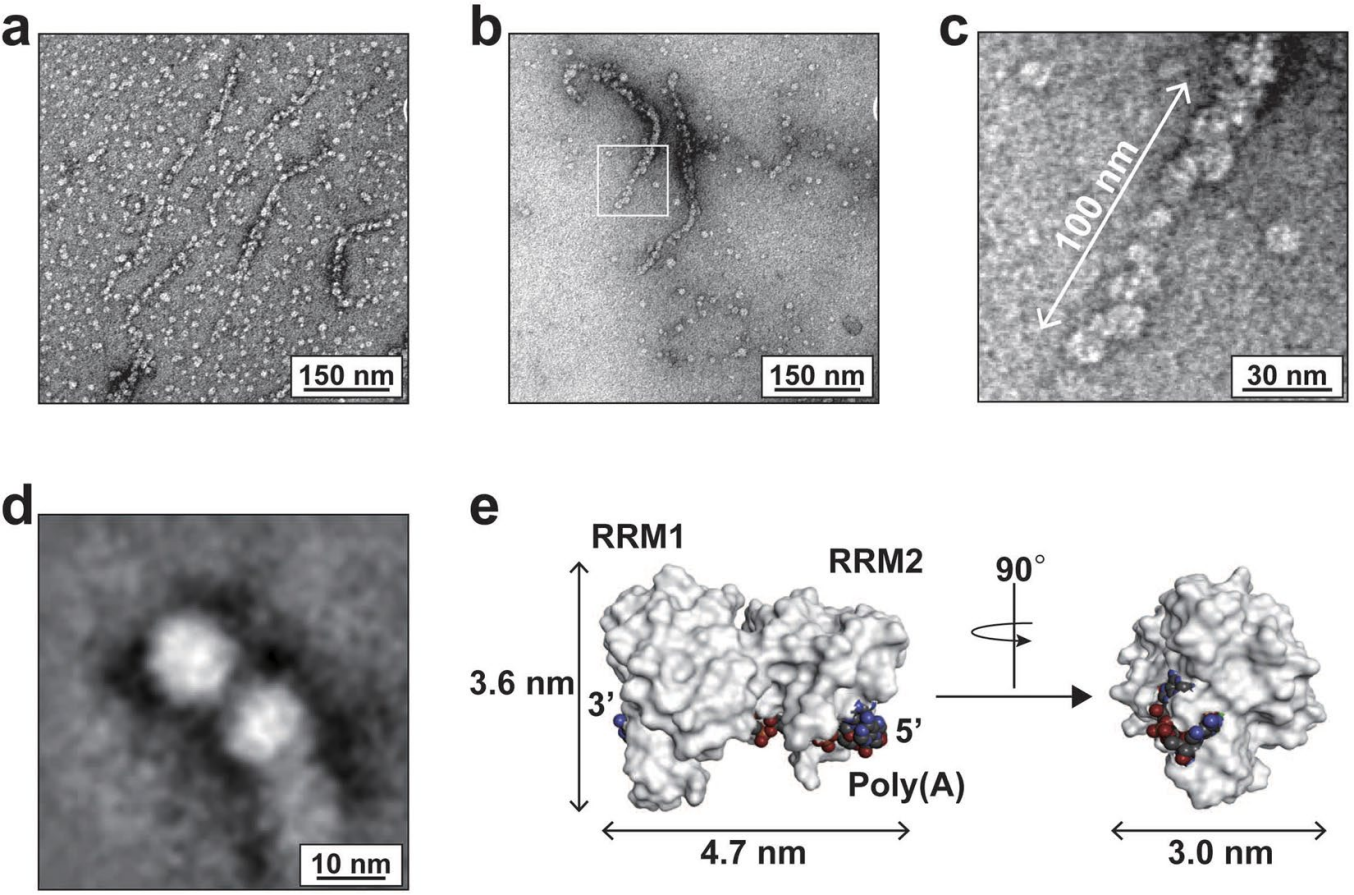
TEM images of poly(A)-bound PABPC1. (**a**,**b**) TEM images of the negatively stained samples. (**c**) Enlarged TEM image of the white box region in (**b**). (**d**) The averaged TEM image. (**e**) Size of the A_9_-bound RRM1/2. A_9_ and RRM1/2 (PDB 1CVJ^37^) are drawn as CPK and surface representations, respectively.

To characterize how the PABPC1 molecules associate with poly(A), we observed negatively stained poly(A)-bound PABPC1 using a transmission electron microscope (TEM). Figure 2a,b clearly show segmented, wormlike structures. In the enlarged image in Fig. 2c, a number of particles is linearly arrayed. The two-dimensional class averaged image of PABPC1-poly(A) complex resulted in a clearer image shown in Fig. 2d, providing an estimation of the particle diameter of 10–12 nm. Considering the size of the RRM1/2-A_9_ complex as 5 nm based on its crystal structure (Fig. 2e), one particle of the TEM image is likely to contain one or a few molecules of poly(A)-bound PABPC1, each of which consists of RRM1/2/3/4 in the poly(A)-bound state, a linker, and PABC.

### Chemical cross-linking of the poly(A)-bound PABPC1 molecules

We further characterized the PABPC1 molecules on poly(A) by chemical cross-linking experiments. As one PABPC1 molecule reportedly covers 23–27 bases of poly(A)^6,11^, we prepared poly(A) RNAs comprising A_24_, A_48_, and A_72_, corresponding to the lengths that are covered by one, two, and three PABPC1 molecules. In addition, a long poly(A), in which the length of 70% of the poly(A) ranges from 150 to 500 bases, was prepared. The concentration corresponding to a 24-base unit of poly(A), *c*(A_24_), was adjusted to 1.0 μM in all the samples, which is half of the PABPC1 concentration of 2.0 μM.

**Figure 3:**
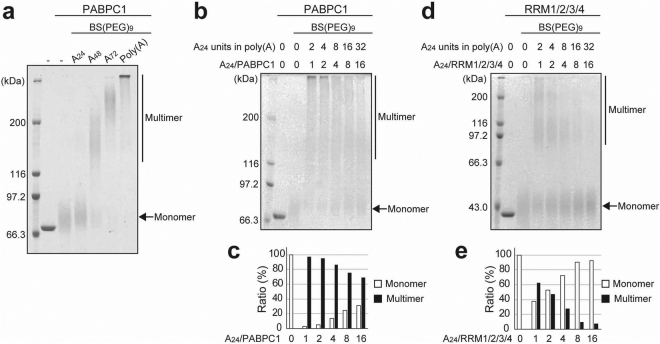
Chemical cross-linking of PABPC1 on poly(A). (**a**) SDS-PAGE analysis of PABPC1 after treatment with the cross-linking reagent, BS(PEG)_9_, in the presence or absence of A_24_, A_48_, A_72_, or long poly(A) ranging from 150–500 bases. (**b**–**e**) Chemical cross-linking of PABPC1 and RRM1/2/3/4 at each molar ratio of a 24-base unit in poly(A) over protein. SDS-PAGE analysis of the cross-linked PABPC1 (**b**) and RRM1/2/3/4 (**d**). The ratios of the monomer and the multimer at each molar ratio in PABPC1 and RRM1/2/3/4 are plotted in (**c**) and (**e**), respectively. The monomer and multimer ratios were calculated by dividing the band intensity of the monomer and the multimer by the total intensity.

We used a cross-linker, BS(PEG)_9_, which cross-links two amino groups of the lysine sidechains. As shown in Fig. 3a, the proteins were observed as a smear in the presence of BS(PEG)_9_, probably owing to heterogeneous chemical modification. The smear probably occurred because different numbers of the lysine residues on the molecular surface of PABPC1 were modified by BS(PEG)_9_. The molecular masses of the smears were 70–90, 140–200, and 210–260 kDa, in the presence of A_24_, A_48_, and A_72_, respectively, and there was a much larger mass for the long poly(A). This correlation between the size of the cross-linked molecules and the length of the poly(A) strongly suggests that multiple PABPC1 molecules on the same poly(A) chain form a one-dimensional multimer, which is consistent with the TEM images shown in Fig. 2.

### Stabilization of the PABPC1 multimer by the intermolecular interactions between the linker-PABC and the RRMs

Whereas the RRM region of PABPC1 is responsible for the poly(A) binding^11^, the intermolecular interactions forming the PABPC1 multimer on poly(A) reportedly involve the linker-PABC region^9,11^. Here, we compared the ability to form a multimer on poly(A) between full length PABPC1 and RRM1/2/3/4, which lacks the linker-PABC region, by chemical cross-linking. It should be noted that the RRM1/2/3/4 prepared here exhibited a binding affinity for poly(A) that is comparable to that of the full length PABPC1 (Table 1 and Supplementary Figure 1b).

We prepared several solutions containing 2.0 μM PABPC1 or RRM1/2/3/4 in the presence of increasing amounts of the long poly(A) wherein the molar ratios of a 24-base unit in the long poly(A) over PABPC1 or RRM1/2/3/4 were 1:1, 2:1, 3:1, 4:1, 8:1, and 16:1. As all the PABPC1 or RRM1/2/3/4 molecules were associated with poly(A) at these concentrations, the increase in the molar ratio of [PABPC1 or RRM1/2/3/4]/[A_24_ unit of poly(A)] leads to a “one-dimensional dilution” of PABPC1 or RRM1/2/3/4 on poly(A).

Figure 3b and d show the SDS-PAGE results after cross-linker treatment, which indicate that RRM1/2/3/4 provides an apparently stronger monomer band as compared to full length PABPC1. The populations of the monomer and the multimer at each poly(A) concentration are plotted in Fig. 3c and e. Whereas approximately 70% of the PABPC1 molecules form a multimer at 16-fold dilution (Fig. 3c), only 63% of the RRM1/2/3/4 molecules form a multimer even at 1-fold dilution, with 8-fold dilution mostly precluding the intermolecular cross-linking of RRM1/2/3/4 (Fig. 3e). These results indicate that the linker and/or PABC regions are crucial for the PABPC1-PABPC1 interactions that are responsible for multimer formation.

As shown in Fig. 3a, however, only poly(A)-bound PABPC1 molecules were cross-linked, whereas no cross-linking was observed in the absence of poly(A), strongly suggesting that multimerization is not due to the mutual intermolecular interaction of the linker-PABC regions that are not involved in the poly(A) binding. Therefore, the direct interactions crucial for the multimer formation are considered to occur between the poly(A)-bound RRM1/2/3/4 region of one PABPC1 molecule and the linker-PABC region of another.

### A middle portion of the linker region (residues 428–442) is responsible for the interaction with poly(A)-bound RRM1/2/3/4

**Figure 4:**
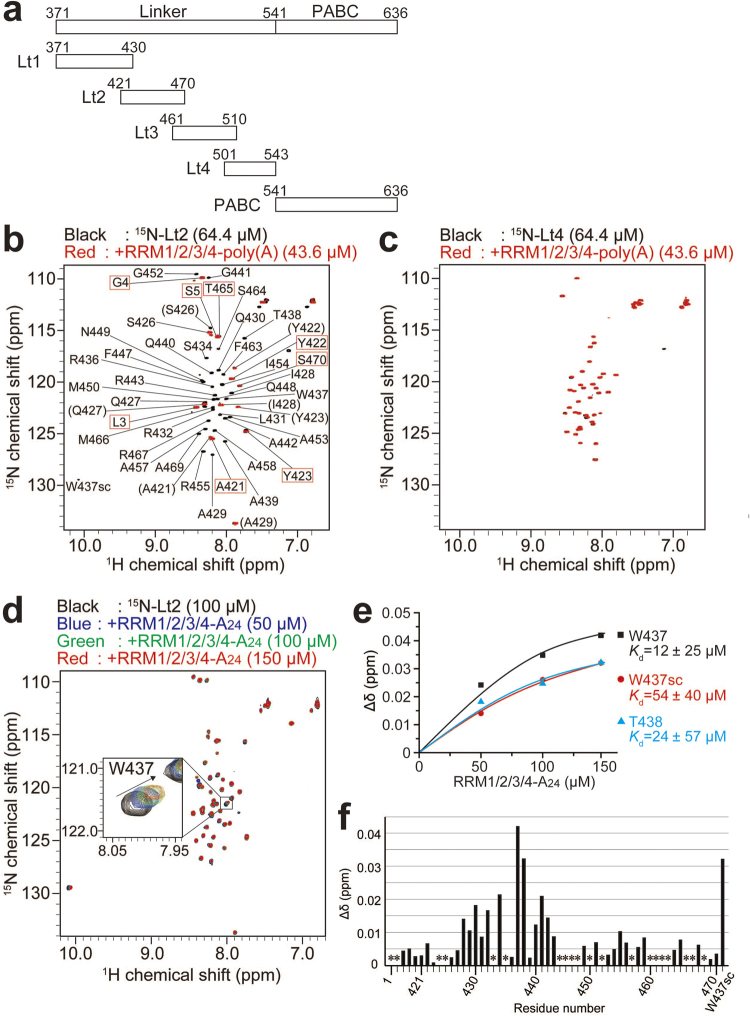
^1^H-^15^N HSQC spectra of Lt2 and Lt4 in the presence and absence of RRM1/2/3/4-poly(A). (**a**) Fragments of the linker-PABC region used in this study. (**b**,**c**) ^1^H-^15^N HSQC spectra of ^15^N-labeled Lt2 (**b**) and Lt4 (**c**) at 64.4 μM, in the presence (red) and absence (black) of 43.6 μM RRM1/2/3/4-poly(A). The peak labels in the parentheses in panel (b) represent the minor peaks of the split signals. The 9 NMR signals with peak labels enclosed by the red box in the panel (b) did not disappear upon addition of RRM1/2/3/4-poly(A). (**d**) ^1^H-^15^N HSQC spectra of ^15^N-labelled Lt2 at 100 μM in the presence and absence of RRM1/2/3/4-A_24_ at 50 (blue), 100 (green) and 150 μM (red). The NMR signal of W437 in the black box is enlarged. (**e**) The estimation of the dissociation constant (*K*_d_). The chemical shift changes of W437 sidechain (sc), W437 and T438 were plotted against RRM1/2/3/4-A_24_ concentration in ^1^H-^15^N HSQC spectra of ^15^N-labelled Lt2 at 100 μM in the presence and absence of RRM1/2/3/4-A_24_ at 50, 100 and 150 μM. The *K*_d_ values were estimated by the curve fitting of the chemical shift changes of the NMR signals for W437sc, W437 and T438, by a 1:1 binding model using the formula,${\rm{\Delta }}{\rm{\delta }}={\rm{\Delta }}{\rm{\delta }}\,\max \,\times \frac{({\rm{X}}+[{\rm{Lt}}2]+{\rm{Kd}})-\sqrt{{({\rm{X}}+[{\rm{Lt}}2]+{\rm{Kd}})}^{2}-4{\rm{X}}[{\rm{Lt}}2]}}{2[{\rm{Lt}}2]}$(4) where Δδ, Δδ max, X, and [Lt2] are the chemical shift changes, maximum of the chemical shift changes, and the concentrations of RRM1/2/3/4-A_24_, and Lt2 (100 μM), respectively. Curve fitting was performed using Origin 5.0 software. (**f**) The chemical shift changes between 0 and 150 μM RRM1/2/3/4-A_24_ are plotted versus the residues of Lt2. The chemical shift changes were calculated using the equation (3) in the Methods. *Indicates Pro or residues without assignments.

To identify which portion of the linker-PABC region is responsible for the intermolecular interactions with poly(A)-bound RRM1/2/3/4, we observed NMR spectra of a number of ^15^N-labelled peptide fragments of PABPC1 containing a portion of the linker and PABC (residues 371–430, referred to as Lt1; residues 421–470, Lt2; residues 461–510, Lt3; residues 501–543, Lt4; residues 541–636, PABC) (Fig. 4a), in the absence or presence of unlabeled RRM1/2/3/4 in complex with long poly(A) RNA with a length of 300 to 5,000 bases. Owing to the large size of the multiple molecules of RRM1/2/3/4 bound to the long poly(A), severe line-broadening of the NMR signals of the ^1^H-^15^N NSQC spectrum was expected upon association of the ^15^N-labelled linker or PABC with the huge complex of RRM1/2/3/4-poly(A).

Figure 4 and Supplementary Figure 2 show the superposition of the spectra of each PABPC1 region in the absence (black) and presence (red) of RRM1/2/3/4-poly(A). In Fig. 4b, 33 NMR signals for Lt2 exhibited significant line broadening upon addition of RRM1/2/3/4-poly(A), whereas only 9 NMR signals remained observed (Fig. 4b), clearly indicating that Lt2 binds to the poly(A)-bound RRM1/2/3/4. In contrast, the NMR signals for Lt1, Lt3, Lt4, or PABC showed no or subtle spectral changes upon addition of RRM1/2/3/4-poly(A) (Fig. 4c, and Supplementary Figure 2). This indicates that Lt2 contains a region that associates with poly(A)-bound RRM1/2/3/4.

NMR resonance assignments of the Lt2 signals (Supplementary Table 1) revealed that the signals for most Lt2 residues (residues 427 to 470) except for the N and C terminal 9 residues were broadened (Fig. 4b), presumably owing to the increase in the rotational correlation time upon binding to the huge RRM1/2/3/4-poly(A) complex. To identify interacting Lt2 residues while avoiding severe line-broadening, we performed NMR titration experiments using a RRM1/2/3/4-A_24_ complex, which possesses a much smaller molecular weight than the long poly(A)-bound multiple RRM1/2/3/4. Figure 4d shows an overlay of a series of ^1^H-^15^N HSQC spectra of ^15^N-labelled Lt2 upon the sequential addition of RRM1/2/3/4-A_24_. Curve fitting of the chemical shift changes for the signals from W437 and T438 using a 1:1 binding model resulted in the *K*_d_ values ranging from 10 to 54 μM (Fig. 4e). Although the NMR spectral change did not saturate during the titration, the chemical shift differences between free Lt2 and Lt2 (100 μM) in the presence of RRM1/2/3/4-A_24_ (150 μM) plotted in Fig. 4f indicate that the signal for W437 exhibited the largest chemical shift change, and that the 15-residue portion from I428 to A442 exhibited significant chemical shift changes. These data strongly suggest that the a 15-residue portion containing W437 is responsible for binding to RRM1/2/3/4 in the poly(A)-bound PABPC1. It should be noted that no typical secondary structure was predicted for Lt2 by its chemical shift values^27–30^ (Supplementary Figure 3).

### RRM2 interacts with Lt2 on poly(A)

**Figure 5:**
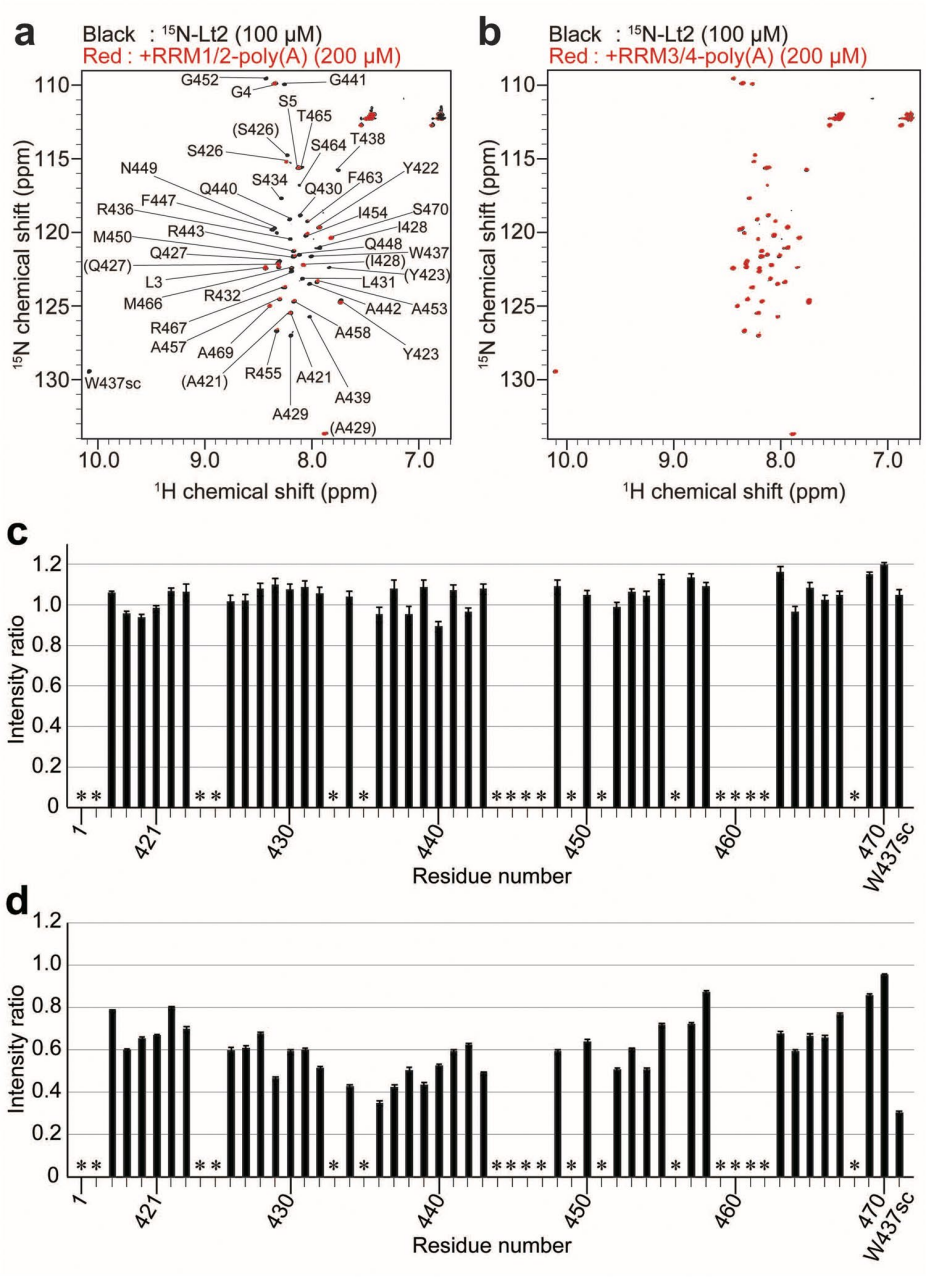
Identification of the RRM domain interacting with Lt2. (**a**,**b**) ^1^H-^15^N HSQC spectra of ^15^N-labelled Lt2 (100 μM) in the presence (red) and absence (black) of RRM1/2-poly(A) (200 μM) (**a**), and of RRM3/4-poly(A) (200 μM) (**b**). The peak labels in the parentheses in panel (a) are for the minor peaks of the split signals. (**c**,**d**) PRE results of ^15^N-labelled Lt2 in the presence of the A_24_-bound, spin-labelled RRM1/2/3/4. The intensity ratios of the ^15^N-Lt2 NMR signals are plotted in the presence of E29C‐MTSL (**c**) and D117C-MTSL (**d**). The intensity ratios were calculated by dividing the signal intensity of the radical (paramagnetic) form MTSL by the signal intensity of the reduced (diamagnetic) form of MTSL. *Indicates Pro or residues without assignments.

To identify which RRM domain binds to the linker region on poly(A), ^1^H-^15^N HSQC spectra of ^15^N-labelled Lt2 were observed in the presence of RRM1/2 or RRM3/4 in complex with long poly(A) (Fig. 5). Significant line broadening of the Lt2 signals was observed in the presence of RRM1/2-poly(A) (Fig. 5a) but not that of RRM3/4-poly(A) (Fig. 5b), indicating that Lt2 binds to the RRM1/2 region of poly(A)-bound PABPC1.

We then tried to identify which of RRM1 or RRM2 acts as the linker binding domain. As the binding affinity of RRM1 or RRM2 for poly(A) is not sufficiently high to form a stable complex with poly(A), we evaluated the intermolecular paramagnetic relaxation enhancement (PRE) effects on Lt2 of a spin label introduced to RRM1 or RRM2 in RRM1/2/3/4-poly(A). When some of the residues of ^15^N-labelled Lt2 approaches within 20 Å of the spin-labelled residue on RRM1 or RRM2 in A_24_-bound RRM1/2/3/4, NMR signals of the Lt2 residues are broadened and reduce their intensities by the PRE effect, leading to the identification which of RRM1 or RRM2 bind to Lt2 (Supplementary Figure 4a). It should be noted that the residues to which the spin labels (MTSL) are chemically attached (E29C in RRM1 or D117C in RRM2) exist at the corresponding positions in each RRM (Supplementary Figure 4b).

We observed HSQC spectra of ^15^N-labelled Lt2 in the presence of the A_24_-bound, spin-labelled RRM1/2/3/4, in both the radical (paramagnetic) form and reduced (diamagnetic) form of MTSL. The PRE effects were evaluated by the signal intensity ratios in the presence of the radical (paramagnetic) form and the reduced (diamagnetic) form, by assuming constant intrinsic *R*_2_ values among all the Lt2 residues. If the spin label of the radical form of MTSL lay within approximately 20 Å from the amide group of Lt2, the amide NMR signal of Lt2 would be broadened by the PRE effect, thus reducing signal intensity.

Figure 5c shows that almost no intensity reduction by PRE was observed following use of the spin-labelled RRM1. Conversely, significant intensity reductions (<0.5) were observed for the signals of A429, S434, R436, W437, A439, and R443 in the sample containing spin-labelled RRM2, representing the PRE effects on these residues (Fig. 5d). Based on these results, we concluded that the linker residues in the Lt2 region bind to RRM2.

### The Lt2 region plays an important role in PABPC1 multimerization on poly(A)

We next investigated the role of the interactions of the linker region in the multimerization of PABPC1. Chemical cross-linking experiments were performed with a deletion mutant of PABPC1 that lacks the Lt2 region (PABPC1ΔLt2) as well as for another mutant that lacks the Lt4 region (PABPC1ΔLt4) as a control, in the same manner as shown in Fig. 3b–e.

**Figure 6:**
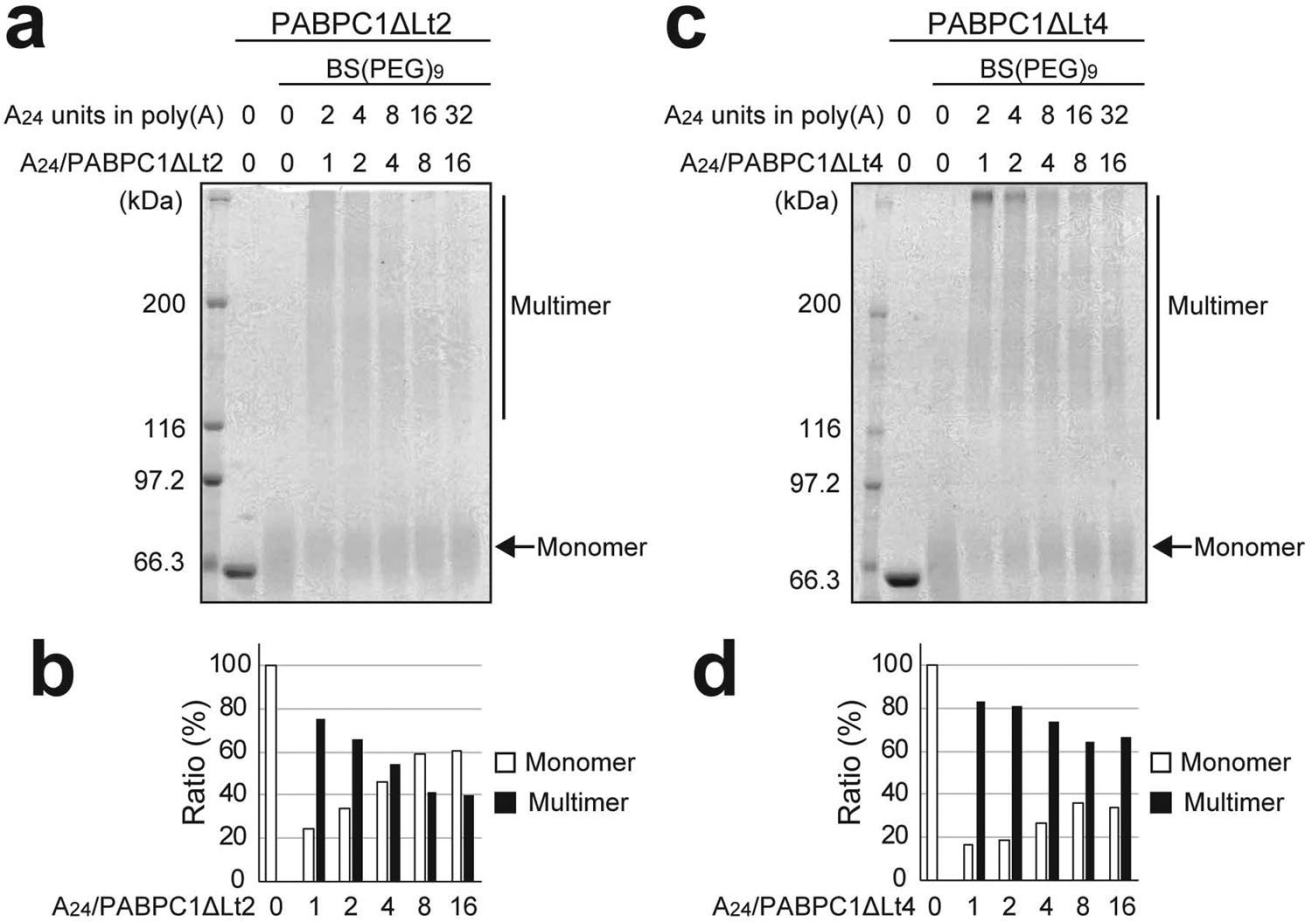
Chemical cross-linking of the poly(A)-bound PABPC1ΔLt2 and PABPC1ΔLt4. (**a**,**c**) SDS-PAGE analysis of the PABPC1**Δ**Lt2 (**a**) and PABPC1**Δ**Lt4 (**c**) proteins after BS(PEG)_9_ treatment at each poly(A) concentration. The ratios of the monomer and the multimer at each molar ratio for PABPC1**Δ**Lt2 and PABPC1**Δ**Lt4 are plotted in (**b**) and (**d**), respectively. The monomer and multimer ratios were calculated by dividing the band intensity of the monomer and the multimer by the total intensity.

Figure 6a and c show the results and Fig. 6b and d show the population plots for PABPC1ΔLt2 and PABPC1ΔLt4, respectively. For PABPC1ΔLt2, dilutions of 1, 4, and 16-fold on poly(A) resulted in the approximate multimer populations of 75, 54, and 40% (Fig. 6b). These values are lower than those for PABPC1 (97, 86, and 69%, respectively, Fig. 3c), but markedly higher than those of RRM1/2/3/4 (63, 28, and 7%, respectively, Fig. 3e). Conversely, the value for PABPC1ΔLt4 were 83, 74, and 67%, respectively (Fig. 6d), which are comparable to those for PABPC1.

These results indicate that the Lt2 region largely contributes to the formation of the PABPC1 multimer on poly(A). As deletion of the Lt4 region, which is the same length as Lt2, showed much less effect on the multimerization, truncation of the linker itself did not impact the multimer formation. These data indicate that the residues in the Lt2 region identified by NMR analyses play an important role in the multimer formation of PABPC1 on poly(A).

### PABPC1 multimerization is important for cap/poly(A)-dependent translation

It has been reported that PABPC1 on the poly(A) tail of mRNA enhances translation in a cap/poly(A)-dependent manner^31^. Here, we investigated the effects of PABPC1 multimerization on cap/poly(A)-dependent translation by using PABPC1ΔLt2 as a multimerization-deficient mutant in a cell-free translation system with a nuclease-treated rabbit reticulocyte lysate (RRL).

**Figure 7:**
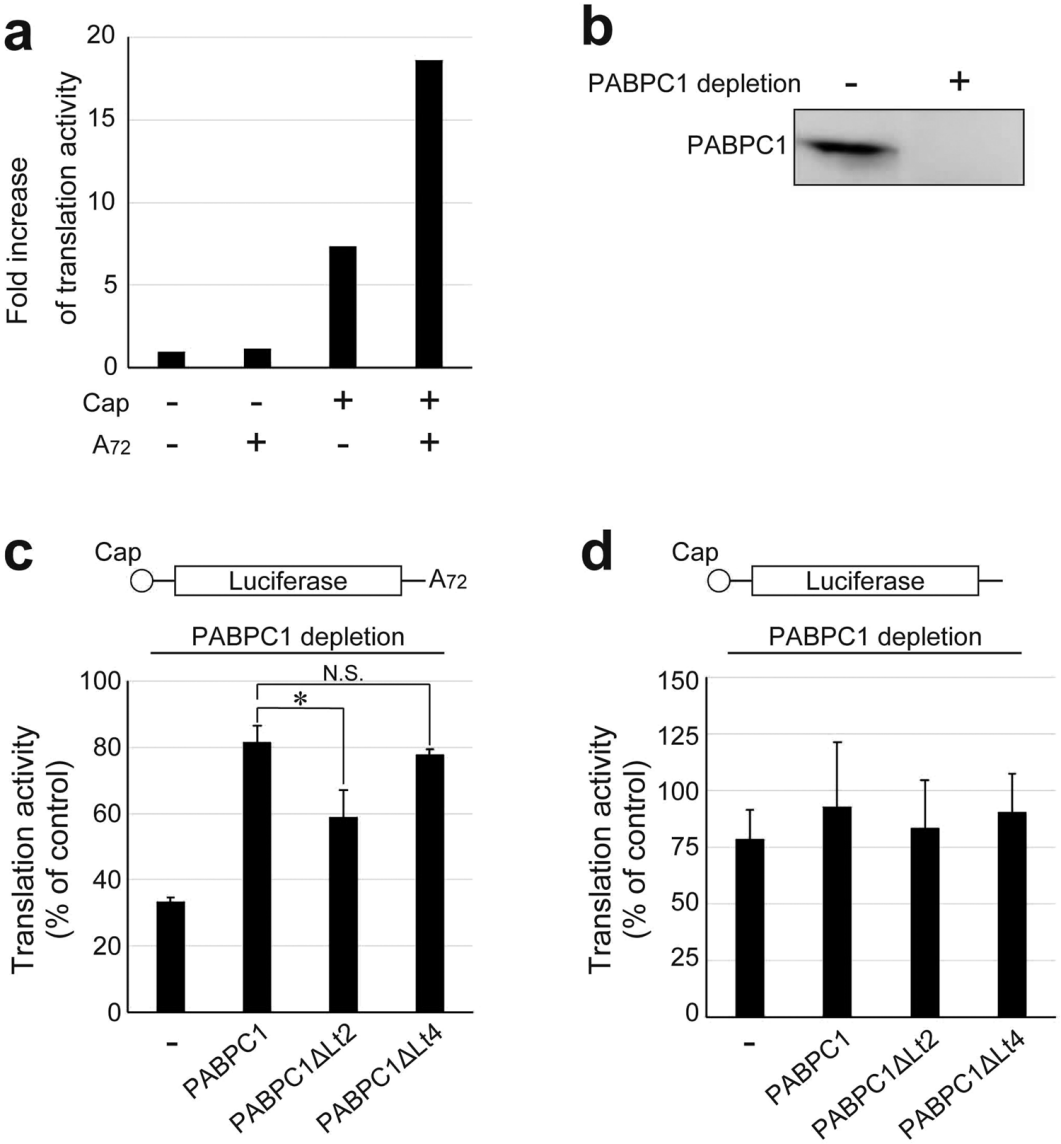
Effects of Lt2 deletion in PABPC1 on cap/poly(A)-dependent translation. (**a**) Translational enhancement by the cap and poly(A) tail. The relative increase of translation activity is shown as the fold increase in the luciferase activity of the mRNA without cap and poly(A). (**b**) Western blotting against PABPC1 for the control RRL and the PABPC1-depleted RRL. The control RRL was treated with GST immobilized glutathione-sepharose beads. The uncropped original blots were shown in Supplementary Figure 6. (**c**,**d**) Translation activity of the capped luciferase mRNA with (**c**) or without (**d**) poly(A) in the PABPC1-depleted RRL in the presence of the purified PABPC1, PABPC1**Δ**Lt2 or PABPC1**Δ**Lt4 (10 nM). Translation activity is shown as a percentage of the luciferase activity in the control RRL. The results represent average values and standard deviations in three independent experiments. *and N.S. indicate *P* < 0.05 and not significant in statistical analysis, respectively.

The translation system established here exhibited significant enhancement of translation in the presence of both cap and poly(A), whereas almost no enhancement was observed in the absence of a 5′-cap (Fig. 7a). Next, we depleted endogenous PABPC1 by using GST-Paip2 (Fig. 7b)^31^, and then added PABPC1ΔLt2, which possesses impaired multimerization activity. Addition of full length PABPC1 or PABPC1ΔLt4 rather than PABPC1ΔLt2 was also performed as control experiments.

Depletion of endogenous PABPC1 reduced the translation of mRNA with a cap/poly(A) to 1/3 of the rate before the depletion. Conversely, the addition of full-length PABPC1 enhanced the translation by 2.5 fold, representing 80% of that before the depletion, suggesting that the incomplete recovery of translation activity reflects the difference between endogenous rabbit PABPC1 and the recombinant human PABPC1 that was added to the system. Although the addition of PABPC1ΔLt4 exhibited essentially the same enhancement of translation as that from the full-length PABPC1, addition of PABPC1ΔLt2 showed only 1.7-fold enhancement, which is significantly smaller than that obtained with the full-length PABPC1 or PABPC1ΔLt4 (Fig. 7).

In contrast, the translation of mRNA without poly(A), which occurs at approximately 40% the rate of mRNA possessing a poly(A) (Fig. 7c), was reduced to 75% of that following endogenous PABPC1 depletion (Fig. 7d). This reduced translation activity was unchanged upon addition of full-length PABPC1, PABPC1ΔLt2, or PABPC1ΔLt4.

These results indicate that cap/poly(A)-dependent translation is suppressed by deletion of the Lt2 region of poly(A)-bound PABPC1, which is a critical region for PABPC1 multimerization on poly(A). Therefore, the PABPC1 multimer on poly(A) is suggested to play important roles in translation.

## Discussion

To date, the structure of multiple PABPC1 molecules bound to a long poly(A) chain has remained unsolved, owing in part to its huge molecular size and the heterogeneity in poly(A) length and thus in the number of PABPC1 molecules bound to the poly(A). Here, we first visualized the PABPC1-poly(A) complex as a TEM image, in which several PABPC1 molecules are linearly arrayed, forming a wormlike structure (Fig. 2).

This one-dimensional array is consistent with the chemical cross-linking results showing a graded increase in the size of the cross-linked PABPC1 molecules in response to the increase in the number of the A_24_ units in poly(A), which is the length of poly(A) recognized by a single PABPC1 molecule^6,11^ (Fig. 3a). Furthermore, the chemical cross-linking experiments with the one-dimensional dilution of PABPC1 on poly(A) clearly indicated that the poly(A)-bound PABPC1 molecule interacts with the PABPC1 molecules on the same poly(A) chain (Fig. 3b).

The analyses for the NMR spectral changes revealed that the intermolecular interactions responsible for PABPC1 multimerization occur between RRM2 of one molecule and a 15-residue portion of the linker region containing W437 (residues 428–442) of another PABPC1 molecule on poly(A) (Figs 4 and 5). The largest chemical shift change was observed for W437, and the largest intensity reduction by PRE from the spin label on D117 in RRM2 was observed for the sidechain of W437. Furthermore, the Lt2 region possesses no typical secondary structure in the free state, as predicted by chemical shift values (Supplementary Figure 3). Therefore, it is strongly suggested that W437 plays the most important role in the multimerization of PABPC1 on poly(A). The PABPC1 mutant lacking the Lt2 region (PABPC1ΔLt2) exhibited markedly less ability to form a multimer (Fig. 6), confirming that the Lt2 region contains a portion contributing to multimer formation.

To date, several crystal and NMR structures of RRM from other proteins in complex with a Trp-containing peptide have been reported, where a Trp sidechain is inserted into a cleft formed by two helices of RRM on the surface of RRM that is opposite to the RNA-binding surface^32–36^ (Supplementary Figure 5a). In the PABPC1 linker region, W437 is the only Trp residue, which seems to be why the W437-containing Lt2 region is critical for the multimer formation of PABPC1 on poly(A). It should be noted that the Trp-binding site of the RRM from other proteins correspond to the eIF4G binding site on RRM2 of PABPC1 (Supplementary Figure 5b), which is proximal to D117 that is spin-labelled in the PRE experiment.

**Figure 8:**
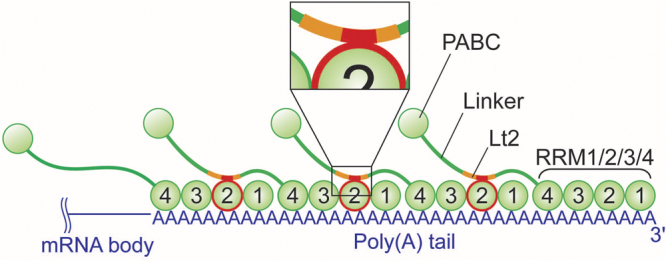
Schematic representation of the repeating nature of the PABPC1 multimer on poly(A). PABPC1 molecules on poly(A) are multimerized by interaction between the RRM2 of one molecule and the Lt2 region containing a portion containing residues 428–442 in linker of the neighbouring molecule. Linker was drawn as a green line. The Lt2 region and the residues 428–442 were colored orange and red, respectively. It should be noted that RRM1/2/3/4 covering poly(A) is illustrated in a linear fashion, as the structure of RRMs 3 and 4 in the poly(A)-bound state remains unknown.

Based on the results of cross-linking showing one-dimensional array, and NMR results demonstrating that the intermolecular interactions between RRM2 and the Lt2 region, particularly, residues 428–442 that contribute to the multimer formation, a schematic of the repeating nature of the PABPC1 multimer is shown in Fig. 8. The direction of RRM1/2/3/4 to poly(A) is given according to that of RRMs 1 and 2 to poly(A) in the crystal structure^37^, where RRMs 1 and 2 bind to the 3′ and 5′ sides of poly(A), respectively. Although RRM1/2/3/4 might adopt certain structure in complex with poly(A), it is depicted in a linear fashion, as the structure of RRMs 3 and 4 in the poly(A)-bound state remains unknown.

The *K*_d_ value for binding of Lt2 to RRM1/2/3/4-A_24_ was estimated to be less than 100 μM, where a protein concentration as high as 1 mM is necessary to saturate the binding. However, as the Lt2 is tethered to the C-terminus of RRM4, the apparent Lt2 concentration is markedly high in the periphery of the RRM4. Considering the 70-residue long unstructured region tethering the Lt2 region to the C-terminus of RRM4, with a reported separation of 7.0 nm^38^, the apparent concentration of the Lt2 existing within a sphere with a radius of 7.0 nm can be estimated as 1.2 mM, based on the following formula:

1 $$\{1\,{\rm{molecule}}/(6.02\times {10}^{23})\}/\{(4\pi /3)\,{(7.0\times {10}^{-9})}^{3}\times {10}^{3}\}=1.2\,{\rm{mM}}$$

This apparent high concentration of the Lt2 region of poly(A)-bound PABPC1 enables the Lt2 region to bind to the RRM2 in the neighbouring PABPC1 bound to the same poly(A) chain, and thus to form a multimer.

When the wild-type PABPC1 was substituted in the RRL by PABPC1ΔLt2, which possesses an impaired ability to form a multimer on poly(A), translation was effectively suppressed (Fig. 7). This result suggests that PABPC1 multimerization is important in the translation system. It has been reported that translation is further enhanced as the length of the poly(A) increases^39^, which appears to be related to the increase in the number of PABPC1 molecules in the multimer bound to the poly(A) chain.

PABPC1 is known to bind a wide range of proteins such as the translation initiation factor eIF4G^14^, translation termination factor eRF3^15^, PABP-interacting protein 1 (Paip1)^16^ and 2 (Paip2)^17^ that enhance and suppress translation, subunits in the deadenylase complex Tob-Caf1^19^, and Pan2-Pan3^20^. The respective binding sites have also been identified: RRM2 for eIF4G, PABC for eRF3 and deadenylase complexes, and RRMs and PABC for Paip1 and Paip2. It should be noted that to our best knowledge, no factors have yet been reported to bind to the Lt2 region.

Although it remains unknown how PABPC1 multimerization enhances translation, it is likely to increase the binding affinity for PABPC1-binding proteins that affect the translation activity. In the multimer, the multiple binding sites of a particular target protein are exposed on the multimer surface at locally high concentrations, which enhances the rebinding of proteins that dissociate from one of the PABPC1 molecules. This effect decreases the apparent *k*_off_ value, through the rebinding of the dissociated molecules to the neighbouring sites, resulting in an increase in binding affinity. Accordingly, in our previous report, we showed that the binding affinity of a PABC-binding peptide derived from eRF3 for PABC of the multiple PABPC1 molecules bound to a long poly(A) chain was 50-fold higher than that for a monomeric PABC^26^.

Notably, proteases from polio virus^40,41^, Coxsackie virus^42^, and HIV^43^ digest the linker region of PABPC1, resulting in the removal of PABC, which is the binding site for translational factors such as eRF3. Our results suggest that the digestion of the linker by the viral proteases significantly impairs the capacity for multimerization, which appears to represent another mechanism underlying the translation suppression mediated by these viruses.

In summary, we have physicochemically characterized the structure of multiple PABPC1 molecules bound to a long poly(A) chain as constituting a one-dimensional wormlike form. We have identified the interactions responsible for PABPC1 multimerization, which occur at residues in the linker region and the RRM2, and shown that such interaction is important for translational activity. The molecular basis of the interactions related to the poly(A)-bound PABPC1 multimer revealed here contributes to an understanding how the various PABPC1-binding proteins regulate translation and mRNA degradation on the poly(A)-bound PABPC1.

## Methods

### Protein expression and purification

The DNA sequence encoding full length human PABPC1 (residues 1–636) and the following mutants thereof were cloned into the pET-42b(+) vector (Novagen, Madison, WI, USA): RRM1/2/3/4 (residues 1–370), RRM1/2 (residues 1–181), RRM3/4 (residues 191–370), PABC (residues 541–636), PABPC1ΔLt2 (residues 1–420, 471–636), and PABPC1ΔLt4 (residues 1–500, 544–636).

The following mutants of human PABPC1 were cloned into the pGEX-6p-1 vector (GE Healthcare, Little Chalfont, UK); Lt1 (residues 371–430), Lt2 (residues 421–470), Lt3 (residues 461–510), and Lt4 (residues 501–543). All of the mutants were generated by PCR-mediated site-directed mutagenesis and confirmed by DNA sequence analysis.

Protein purification details are described in the Supplementary methods. Briefly, the GST-fusion proteins were expressed in *Escherichia coli* cells and purified using a Glutathione-Sepharose 4B column (GE Healthcare), followed by digestion by factor Xa (Novagen) or PreScission Protease (GE Healthcare). The cleaved GST and the non-cleaved fusion proteins were removed by using a Glutathione-Sepharose 4B column. The bound nucleic acids were removed via a cation exchange column (HiTrap SP HP, GE Healthcare), followed by gel filtration using HiLoad 16/600 Superdex 75 pg (GE Healthcare).

Lt1, Lt2, Lt3, and Lt4 were further purified by high-performance liquid chromatography (Shimadzu Corp., Kyoto, Japan) using a reverse-phase C18 column (GL Sciences, Tokyo, Japan). The molecular weights of Lt1, Lt2, Lt3, and Lt4 were confirmed by MALDI-TOF mass spectrometry, using an AXIMA-CFR Plus mass spectrometer (Shimadzu Corp.).

Uniformly ^15^N or ^13^C, ^15^N-double labelled proteins for NMR experiments were prepared by growing *E. coli* host cells in M9 minimal medium containing ^15^NH_4_Cl or ^15^NH_4_Cl and ^13^C_6_-glucose.

### Preparation of poly(A)

Poly(A) RNAs with 24, 48, and 72 bases (hereafter, referred to as A_24_, A_48_, and A_72_) were purchased from Gene Design, Inc. (Osaka, Japan). Long poly(A) RNAs of varying lengths from 300 to 5,000 bases were purchased from GE Healthcare. Poly(A) RNAs with a medium length from 150 to 500 bases were purchased from Sigma-Aldrich (St. Louis, MO, USA). Poly(A) RNAs were boiled at 65 °C for 3 min and cooled rapidly on ice prior to initiating experiments.

For TEM, the poly(A) corresponding to an approximate length of 200 bases was extracted from the denaturing gel of the poly(A)s with a medium length, and separated by electrophoresis using a denaturing gel with 8 M urea and 8% polyacrylamide (w/v) (monomer:bis = 19:1).

It should be noted that in this study, the molar concentrations of the long poly(A) are described as those of the 24-base unit, *c*(A_24_), based on the following Lambert-Beer’s law:

2 $$c({{\rm{A}}}_{24})={{\rm{Abs}}}_{260}/{\varepsilon }_{24}\,l$$

where *c*(A_24_) is the molar concentration of the A_24_ unit in the long poly(A), Abs_260_ is the absorption of UV at the wavelength of 260 nm, *ε*_24_ is the molar extinction coefficient value of 364,800 (l/mol·cm) that is 24 times the molar extinction coefficient value of one adenine base, and *l* is the optical path length of 1 cm, respectively.

### TEM analysis

We purified the PABPC1-poly(A) complex by gel filtration chromatography using a Superose 6 increase 5/150 GL column. The applied sample contained 7.0 μM PABPC1 and poly(A) at *c*(A_24_) of 3.5 μM. The running buffer contained 20 mM NaH_2_PO_4_ (pH 7.2), 500 mM NaCl, and 1 mM dithiothreitol (DTT). The eluted fractions containing the PABPC1-poly(A) complex were chemically cross-linked by treatment of 200 mM glutaraldehyde at 4 °C for 30 min.

The samples were adsorbed onto thin carbon films supported by copper mesh grids, which were rendered hydrophilic in advance by glow discharge under low air pressure. The PABPC1-poly(A) complexes were negatively stained with EM stainer (Nisshin EM, Tokyo, Japan), blotted, and dried in air. Samples were observed using a JEM1230 transmission electron microscope (JEOL, Tokyo, Japan) at an acceleration voltage of 100 kV. Images were recorded using a TVIPS F114T CCD camera (Oslo, Norway).

For 2D classification, a total of 193 boxed images were manually extracted from 39 independently recorded CCD images with 128 by 128 pixel size using BOXER software in EMAN2^44^. We applied eight rounds of reference free 2D-class averaging using EMAN2 and they were classified into six classes.

### Chemical cross-linking

In the presence of 2.0 μM full-length PABPC1, poly(A) with a fixed length, i.e., A_24_, A_48_, and A_72_, was prepared at 1.0, 0.5, and 0.33 μM, respectively, to adjust the *c*(A_24_) of all poly(A)s to 1.0 μM. In addition, long poly(A)s at a *c*(A_24_) of 1.0 μM were also prepared in the presence of 2.0 μM full-length PABPC1 and mutant solution. These samples were incubated at room temperature for 15 min in a buffer containing 10 mM Na_2_HPO_4_, 1.8 mM KH_2_PO_4_, 137 mM NaCl, 2.7 mM KCl, 5 mM MgSO_4_, and 1 mM DTT at pH 7.4, and incubated with 2 mM bis-*N*-succinimidyl-(nonaethylene glycol) ester (BS(PEG)_9_ (Thermo Fisher Scientific, Waltham, MA, USA) at 4 °C for 1 h. BS(PEG)_9_ was inactivated by addition of 50 mM Tris-HCl. The cross-linked proteins were analysed by sodium dodecyl sulphate-polyacrylamide electrophoresis (SDS-PAGE).

### NMR analyses

Data were collected on Bruker Avance 400, 500, or 600 spectrometers using triple-resonance or cryogenic probes. All spectra were processed using Bruker TopSpin 3.5 software, and the data were analysed with Sparky (T. D. Goddard and D. G. Kneller, Sparky 3, University of California, San Francisco, CA). Titrations of ^15^N-labelled Lt1, Lt2, Lt3, Lt4, and PABC with unlabelled RRM1/2/3/4, RRM1/2, and RRM3/4 in the presence of long poly(A) or A_24_ were monitored by ^1^H-^15^N heteronuclear single quantum coherence (HSQC) spectra at 303 K in a buffer containing 26 mM NaH_2_PO_4_ (pH 6.0), 100 mM NaCl, and 8% ^2^H_2_O.

Chemical shift changes were calculated using the following equation^45^:

3 $${\rm{\Delta }}{\rm{\delta }}=\sqrt{{{\rm{\Delta }}}_{1{\rm{H}}}^{2}+{({{\rm{\Delta }}}_{15{\rm{N}}}/6.5)}^{2}}$$

where Δ_1H_ and Δ_15N_ are the chemical shift changes in the ^1^H and ^15^N dimensions, respectively.

Sequential assignments of the backbone NMR resonances of Lt2 were achieved by HNCACB, CBCA(CO)NH, C(CO)NH, HN(CA)CO, HNCO, and ^15^N-edited TOCSY-HSQC experiments at 303 K in the buffer containing 26 mM NaH_2_PO_4_ (pH 6.0), 100 mM NaCl, 0.5 mM ethylenediaminetetraacetic acid (EDTA), and 8% ^2^H_2_O.

For paramagnetic relaxation enhancement (PRE) analysis, the E29C or D117C mutant of Cys-less RRM1/2/3/4 (C43S, C128S, C132S, and C339S of RRM1/2/3/4) was cloned into the pGEX-6p-1 vector and purified in the same manner as PABPC1. These purified proteins were incubated overnight with 10 mM DTT at 4 °C. After removal of DTT by dialysis, a 10-fold excess of (1-oxyl-2,2,5,5-tetramethylpyrroline-3-methyl) methanethiosulphonate (MTSL) was added to introduce a spin label onto the protein. The samples were incubated overnight at 4 °C in the dark. The MTSL modification was confirmed by mass spectroscopy. ^1^H-^15^N HSQC spectra of ^15^N-labelled Lt2 (100 μM) with the MTSL-modified protein (40 μM) and A_24_ (40 μM) were acquired in the presence and absence of 2 mM ascorbate at 288 K in a buffer containing 20 mM KH_2_PO_4_ (pH 6.0), 150 mM NaCl, and 10% ^2^H_2_O.

### *In vitro* translation assay

Luciferase mRNAs with or without an A_72_ tail were synthesized with T7 RNA polymerase after linearization of pBK-5F-Luc-pA with BsmBI or XbaI, respectively. pBK-5F-Luc-pA was generated by inserting the following three PCR fragments into the XhoI and ManHI site of pBluescript II SK(-): 5xFlag-tag that was amplified using the primer pair NH733/NH124 and pCMV-5xFlag^46^ as a template, luciferase cDNA that was amplified using the primer pair NH363/NH373 and pGL4.16 (Promega) as a template, and β-globin 3’UTR that contains 72 nts of poly(A) tail that was amplified using the primer pair NH734/NH770 and pFlag-CMV/TO-BGG^18^ as a template. To synthesize capped mRNAs, 3′-O-Me-m^7^G(5′) pppG (Stratagene, Santa Clara, CA, USA) was used.

The primers used in the PCR reactions were as follows.

NH124: 5′-GATCTATCGATGTCGACGATATCGAATTCAAGCTT-3′ (antisense)

NH363: 5′-CAATCCAAGCTTGTTGGTAAAGCCACCATG-3′ (sense)

NH373: 5′-TGCCTCGAGTTACACGGCGATCTTGCCGCC-3′ (antisense)

NH733: 5′-ACCCTCGAGCCCACCATGGCATCAATGGAT-3′ (sense)

NH734:5′-CTTGAATTCGATATCGTCGACGCTCGCTTTCTTGCTGTCCAATTTCT-3′ (sense)

NH770: 5′-GTCTCT(73)CTAGACATCATTGCAATGAAAA-3′ (antisense).

Human Paip2 was cloned into the pGEX-6p-1 vector. GST-Paip2 was purified using a Glutathione-Sepharose 4B column and an anion exchange column (RESOURCE Q, GE Healthcare) after being expressed in *E. coli* cells.

The *in vitro* translation reaction was performed as described below. Nuclease-treated rabbit reticulocyte lysate (hereafter, referred to as RRL) (7.5 μl) was reconstituted with 7.5 μl of a buffer consisting of 10 mM Hepes-KOH (pH 7.5), 142 mM KCl, 1.32 mM MgCl_2_, 0.1 mM EDTA, 7 mM β-mercaptoethanol, 20 μM complete amino acid mixture (Promega, Madison, WI, USA), 1.6 units/μl recombinant RNase inhibitor (TaKaRa), and 2 μg/ml luciferase mRNA. The endogenous PABPC1 was depleted by GST-Paip2 immobilized on gulutathione-sepharose beads, and the purified PABPC1 or its mutants (10 nM) were added. After these samples were incubated for 1 h at 30 °C, luciferase activity was measured using the Bright-Glo luciferase assay system (Promega).

One-way analysis of variance (ANOVA) followed by the Tukey test was used to evaluate differences among the four groups. Differences were considered to be significant at P < 0.05.

### Data availability

All data generated or analysed during this study are included in this published article and its Supplementary Information files.

## Electronic supplementary material

Supplementary information
