# Supplementary material for: Characterization of the multimeric structure of poly(A)-binding protein on a poly(A) tail
Source: Sci Rep. 2018 Jan 23;8:1455. doi: 10.1038/s41598-018-19659-6 (PMC5780489; doi:10.1038/s41598-018-19659-6)
Supplement: Supplementary file 1 — Supplementary information [file 41598_2018_19659_MOESM1_ESM.pdf]

Characterization of the multimeric structure of poly(A)-binding protein on a poly(A) tail

Ryoichi Sawazaki[1], Shunsuke Imai[2], Mariko Yokogawa[1], Nao Hosoda[3], Shin-ichi Hoshino[3], Muneyo Mio[4], Kazuhiro Mio[4], Ichio Shimada[2] and Masanori Osawa*[1,2]

[1] Graduate School of Pharmaceutical Sciences, Keio University, Shibakoen, Minato-ku, Tokyo 105-8512, Japan

[2] Graduate School of Pharmaceutical Sciences, The University of Tokyo, Hongo, Bunkyo-ku, Tokyo 113-0033, Japan

[3] Graduate School of Pharmaceutical Sciences, Nagoya City University, Tanabe-dori, Mizuho-ku, Nagoya 467-8603, Japan

[4] Molecular Profiling Research Center for Drug Discovery and OPERANDO Open Innovation Laboratory, National Institute of Advanced Industrial Science and Technology, Koto-ku, Tokyo 135-0064, Japan

* Correspondence should be addressed to M.O. (osawa-ms@pha.keio.ac.jp)

Supplementary Information

Supplementary methods

Protein expression and purification

The DNA sequence encoding full length human PABPC1 (residues 1–636) and the following mutants thereof were cloned into the pET-42b(+) vector (Novagen, Madison, WI, USA): RRM1/2/3/4 (residues 1–370), RRM1/2 (residues 1–181), RRM3/4 (residues 191–370), PABC (residues 541–636), PABPC1ΔLt2 (residues 1–420, 471–636), and PABPC1ΔLt4 (residues 1–500, 544–636).

The following mutants of human PABPC1 were cloned into the pGEX-6p-1 vector (GE Healthcare, Little Chalfont, UK); Lt1 (residues 371–430), Lt2 (residues 421–470), Lt3 (residues 461–510), and Lt4 (residues 501–543). These proteins possess a N-terminal 5-residue sequence (Gly-Pro-Leu-Gly-Ser) as a cloning artifact. It should be noted that a Trp residue was inserted between the N-terminal 5-residue sequence and the residue 461 of Lt3, which possesses no Trp residue, to quantify its concentration based on the UV absorbance at 280 nm. All of the mutants were generated by PCR-mediated site-directed mutagenesis and confirmed by DNA sequence analysis.

Transformed *Escherichia coli* cells (BL21-CodonPlus (DE3)-RP, Novagen) were grown in 1 L Luria Bertani medium in the presence of 40 mg kanamycin to an absorbance at 600 nm of 0.6-0.8 at 37 °C. The expressions of glutathione S-transferase (GST)-fusion proteins were induced by addition of 0.5 or 1 mM isopropyl β-D-1-thiogalactopyranoside and cells were grown for 3-6 hours at 37 °C. The cells were harvested by centrifuge and disrupted by sonication in 30 ml lysis buffer (10 mM Na_2HPO_4 (pH 7.4), 1.8 mM KH_2PO_4, 637 mM NaCl, 2.7 mM KCl, 10% glycerol, 1 mM phenylmethylsulfonyl fluoride, 0.5 mM 4-(2-aminoethyl) benzenesulfonyl fluoride hydrochloride, 0.15 μM aprotinin, 1 μM E-64 and 1 μM leupeptin) with 0.15% polyethylenimine for removal of nucleic acids in lysate.

Cell debris was removed by centrifuge and the supernatants containing GST-tagged RRM1/2/3/4, RRM1/2 and RRM3/4 were incubated for 2 hours in the presence of 50 μg/ml DNase (Sigma-Aldrich) at room temperature. The supernatants were applied to Glutathione-Sepharose 4B column (GE Healthcare) equilibrated with the purification buffer (10 mM Na_2HPO_4 (pH 7.4), 1.8 mM KH_2PO_4, 137 mM NaCl, 2.7 mM KCl and 1 mM dithiothreitol (DTT)) at 4 °C, and washed using the purification buffer containing 600 mM NaCl. The GST-fusion proteins were eluted with the elution buffer (100 mM Tris-HCl (pH 7.8), 300 mM NaCl and 20 mM glutathione

(reduced form)), followed by digestion by factor Xa (Novagen) or PreScission Protease (GE Healthcare). The cleaved GST and the non-cleaved fusion proteins were removed by using a Glutathione-Sepharose 4B column.

For removal of the bound nucleic acids, the proteins were applied to a cation exchange column (HiTrap SP HP, GE Healthcare) equilibrated with a buffer containing 10 mM Na_2PO_4 (pH 6.5), 10 mM NaCl and 1 mM DTT and eluted with increasing NaCl concentration. The final A_{260}/A_{280} ratio of PABPC1 was ca. 0.7. The degradation products were removed by gel filtration in the running buffer containing 20 mM NaH_2PO_4 (pH 6.5), 500 mM NaCl and 1 mM DTT using HiLoad 16/600 Superdex 75 pg (GE Healthcare).

SPR analysis

All experiments were conducted at 298 K using a Biacore T200 (GE Healthcare). The running buffer contained 10 mM Na_2HPO_4, 1.8 mM KH_2PO_4, 287 mM NaCl, 2.7 mM KCl, 5.0 mM $MgSO_4$, 1.0 mM DTT, and 5% glycerol at pH 7.4. 5′-biotinylated A_{24} was immobilized at a flow rate of 10 µl/min on a streptavidin-coated sensor chip. The purified full-length PABPC1 and RRM1/2/3/4 were injected at a flow rate of 40 µl/min for 90 sec and the running buffer was flowed over the sensor chip at the same flow rate for 600 sec for dissociation. The surface was regenerated with a 0.10% (w/v) SDS solution. All samples were flowed over a non-immobilized surface in flow cells and that response was used as a reference. The kinetic analysis of data sets was performed using Biacore T200 evaluation software, Version 2.0 (GE Healthcare).

Supplementary Table 1 Chemical shift table of Lt2

Residues		N	H	HA	HB	HB2	HB3	HG	HG1	HG2	HG3	HD	HD1	CA	CB	CG	CG1	CG2	CD	CD1	CD2	CO
G	1	-	-	-	-	-	-	-	-	-	-	-	-	-	-	-	-	-	-	-	-	-
P	2	-	-	-	-	-	-	-	-	-	-	-	-	63.215	32.222	26.940	-	-	49.652	-	-	177.043
L	3	122.447	8.509	4.377	1.681	-	-	-	-	-	-	0.883	-	55.485	42.472	26.780	-	-	-	23.575	24.613	178.023
G	4	109.902	8.418	4.024	-	-	-	-	-	-	-	-	-	45.414	-	-	-	-	-	-	-	174.309
S	5	115.591	8.188	4.420	3.864	-	-	-	-	-	-	-	-	58.534	64.137	-	-	-	-	-	-	-
A	421	125.494	8.274	4.292	1.285	-	-	-	-	-	-	-	-	52.506	19.101	-	-	-	-	-	-	176.747
Y	422	119.654	8.000	4.463	2.869	-	-	-	-	-	-	-	-	57.896	39.172	-	-	-	-	-	-	174.541
Y	423	124.783	7.797	4.741	-	2.976	2.708	-	-	-	-	-	-	54.988	40.520	-	-	-	-	-	-	172.568
P	424	-	-	-	-	-	-	-	-	-	-	-	-	-	-	-	-	-	-	-	-	-
P	425	-	-	-	-	-	-	-	-	-	-	-	-	63.286	31.797	27.261	-	-	-	-	-	177.133
S	426	115.199	8.310	4.420	3.885	-	-	-	-	-	-	-	-	58.534	64.066	-	-	-	-	-	-	-
Q	427	121.984	8.379	4.399	1.981	-	-	2.366	-	-	-	-	-	55.981	29.598	33.762	-	-	-	-	-	176.127
I	428	121.076	8.000	4.107	1.884	-	-	-	-	-	-	-	0.922	61.655	38.676	-	27.261	17.310	-	12.414	-	176.205
A	429	127.069	8.274	4.281	1.403	-	-	-	-	-	-	-	-	52.861	18.960	-	-	-	-	-	-	177.662
Q	430	118.841	8.183	4.302	2.002	-	-	2.377	-	-	-	-	-	55.910	29.456	33.682	-	-	-	-	-	175.857
L	431	123.158	8.161	4.334	1.619	-	-	-	-	-	-	0.879	-	55.485	42.472	23.409	-	-	-	24.613	26.699	177.004
R	432	122.650	8.263	4.655	-	-	-	1.745	-	-	-	3.211	-	53.783	30.165	-	-	-	-	-	-	173.974
P	433	-	-	-	-	-	-	-	-	-	-	-	-	62.577	32.080	27.261	-	-	-	-	-	176.708
S	434	117.673	8.359	4.741	3.846	-	-	-	-	-	-	-	-	56.549	63.624	-	-	-	-	-	-	172.994
P	435	-	-	-	-	-	-	-	-	-	-	-	-	63.428	31.797	27.261	-	-	52.461	-	-	176.914
R	436	120.466	8.274	4.238	-	1.681	1.510	-	-	-	-	3.130	-	56.549	30.591	26.780	-	-	43.452	-	-	176.153

W	437	121.634	8.075	4.752	3.297	-	-	-	-	-	-	-	-	57.471	29.740	-	-	-	-	-	-	176.334
T	438	115.794	7.818	4.238	4.142	-	-	-	1.107	-	-	-	-	61.584	70.308	-	-	21.242	-	-	-	173.845
A	439	125.748	8.092	4.206	1.371	-	-	-	-	-	-	-	-	52.719	18.818	-	-	-	-	-	-	177.830
Q	440	119.095	8.279	4.292	2.013	-	-	-	-	3.029	2.387	-	-	56.336	29.314	33.762	-	-	-	-	-	176.643
G	441	109.902	8.322	3.939	-	-	-	-	-	-	-	-	-	45.201	-	-	-	-	-	-	-	173.664
A	442	123.513	8.092	4.345	1.349	-	-	-	-	-	-	-	-	52.364	19.243	-	-	-	-	-	-	177.495
R	443	121.279	8.241	4.548	1.670	-	-	-	-	-	-	-	-	53.783	30.165	-	-	-	-	-	-	174.245
P	444	-	-	-	-	-	-	-	-	-	-	-	-	-	-	-	-	-	-	-	-	176.424
H	445	119.821	8.415	-	-	-	-	-	-	-	-	-	-	-	-	-	-	-	-	-	-	173.316
P	446	-	-	-	-	-	-	-	-	-	-	-	-	-	-	-	-	-	-	-	-	176.747
F	447	119.857	8.423	4.666	3.115	-	-	-	-	-	-	-	-	57.754	39.102	-	-	-	-	-	-	175.702
Q	448	121.583	8.188	4.292	1.970	-	-	2.270	-	-	-	-	-	55.910	29.740	33.601	-	-	-	-	-	175.134
N	449	119.765	8.416	4.848	2.772	-	-	-	-	-	-	-	-	53.783	39.102	-	-	-	-	-	-	174.683
M	450	121.634	8.247	-	4.805	1.991	-	2.590	-	-	-	-	-	53.428	32.435	-	-	-	-	-	-	174.141
P	451	-	-	-	-	-	-	-	-	-	-	-	-	63.641	31.938	27.181	-	-	-	-	-	177.585
G	452	109.547	8.504	3.939	-	-	-	-	-	-	-	-	-	45.272	-	-	-	-	-	-	-	173.871
A	453	123.361	8.017	4.345	1.392	-	-	-	-	-	-	-	-	52.435	19.385	-	-	-	-	-	-	177.611
I	454	120.263	8.134	4.153	1.845	-	-	-	1.852	-	-	-	0.889	61.083	38.605	-	27.101	17.390	-	-	-	176.063
R	455	126.764	8.402	4.655	1.724	-	-	-	-	-	-	3.211	-	53.783	30.165	-	-	-	-	-	-	173.974
P	456	-	-	-	-	-	-	-	-	-	-	-	-	63.003	32.009	27.261	-	-	52.060	-	-	176.489
A	457	124.580	8.375	4.302	1.392	-	-	-	-	-	-	-	-	52.222	19.243	-	-	-	-	-	-	177.095
A	458	124.732	8.241	4.591	1.371	-	-	-	-	-	-	-	-	50.378	18.250	-	-	-	-	-	-	175.496
P	459	-	-	-	-	-	-	-	-	-	-	-	-	62.849	32.130	-	-	-	-	-	-	176.643
R	460	122.392	8.383	-	-	-	-	-	-	-	-	-	-	53.881	29.871	-	-	-	-	-	-	173.948

P	461	-	-	-	-	-	-	-	-	-	-	-	-	-	-	-	-	-	-	-	-	-
P	462	-	-	-	-	-	-	-	-	-	-	-	-	63.215	31.797	27.261	-	-	52.461	-	-	176.721
F	463	119.247	8.118	4.666	3.147	-	-	-	-	-	-	-	-	57.825	39.456	-	-	-	-	-	-	175.844
S	464	116.809	8.188	4.495	3.842	-	-	-	-	-	-	-	-	58.180	64.208	-	-	-	-	-	-	-
T	465	115.550	8.172	4.342	4.282	-	-	-	1.232	-	-	-	-	62.082	69.713	-	-	21.563	-	-	-	-
M	466	122.449	8.260	4.473	2.013	-	-	2.580		-	-	-	-	55.556	32.931	31.836	-	-	-	-	-	175.753
R	467	123.767	8.327	4.677	1.745	-	-	-	-	-	-	3.211	-	53.995	30.236	-	-	-	-	-	-	174.012
P	468	-	-	-	-	-	-	-	-	-	-	-	-	63.144	32.080	-	-	-	-	-	-	176.553
A	469	125.037	8.466	-	-	-	-	-	-	-	-	-	-	52.648	19.172	-	-	-	-	-	-	176.992
S	470	120.365	7.888	-	3.858	-	-	-	-	-	-	-	-	59.813	65.059	-	-	-	-	-	-	178.629
A	421	125.329	8.303	4.316	1.278	-	-	-	-	-	-	-	-	52.468	19.137	-	-	-	-	-	-	177.076
Y	422	118.483	7.963	4.527	-	3.040	2.847	-	-	-	-	-	-	57.754	39.031	-	-	-	-	-	-	174.270
Y	423	122.396	7.909	4.431	2.883	-	-	-	-	-	-	-	-	55.839	41.448	-	-	-	-	-	-	173.368
S	426	114.778	8.306	4.409	3.896	-	-	-	-	-	-	-	-	58.818	64.137	-	-	-	-	-	-	-
Q	427	122.142	8.391	4.655	1.724	-	-	-	-	-	-	-	-	55.910	29.740	33.682	-	-	-	-	-	175.560
I	428	122.244	8.150	4.180	1.895	-	-	-	-	-	-	-	0.922	61.364	38.818	-	26.940	17.390	-	12.735	-	174.941
A	429	133.720	7.955	4.152	1.334	-	-	-	-	-	-	-	-	53.924	20.492	-	-	-	-	-	-	170.324

Supplementary figures

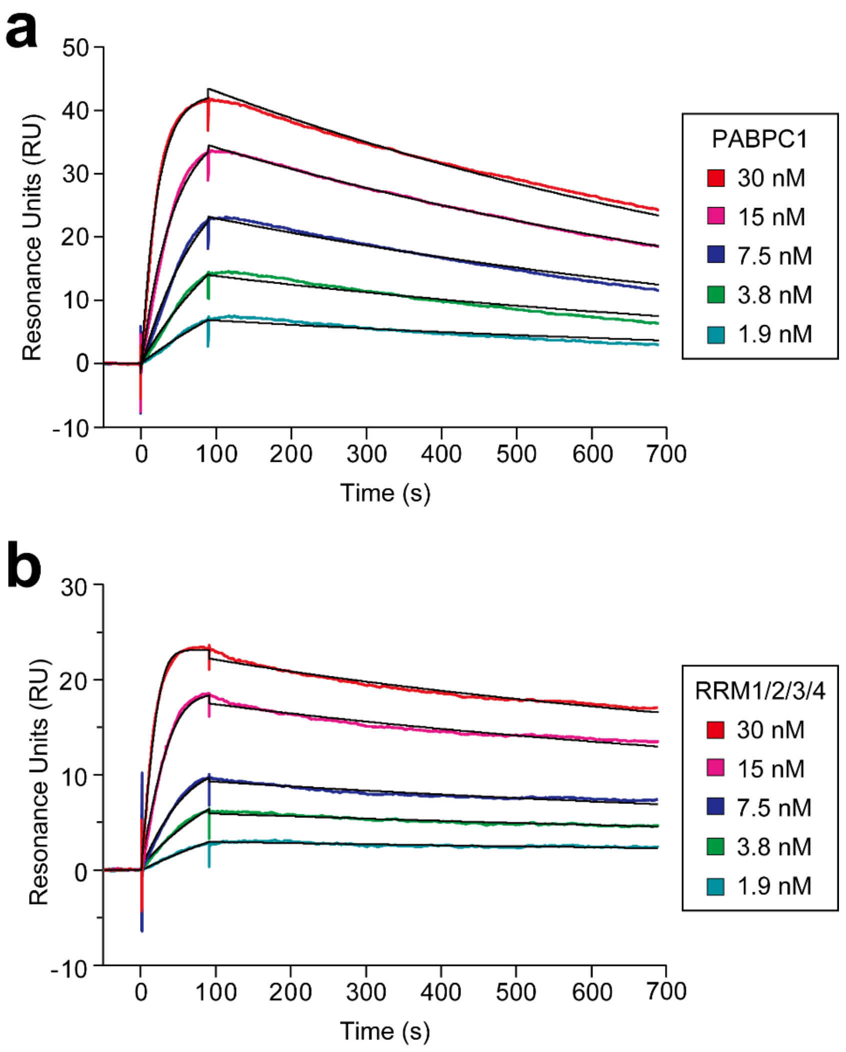

Supplementary Figure 1 SPR analysis for poly(A)-binding affinity of the purified full-length and RRM1/2/3/4 region of human PABPC1

The binding affinities of the prepared PABPC1 and RRM1/2/3/4 for 5′-biotinylated A_{24} immobilized on a streptavidin-coated sensor chip were characterized by SPR. Colour curves are the sensorgrams at each concentration of PABPC1 (a) and RRM1/2/3/4 (b). Curves were successfully fitted using a 1:1 binding model, yielding k_{ass} and k_{dis} values as summarized in Table 1. The fitted curves are shown in black.

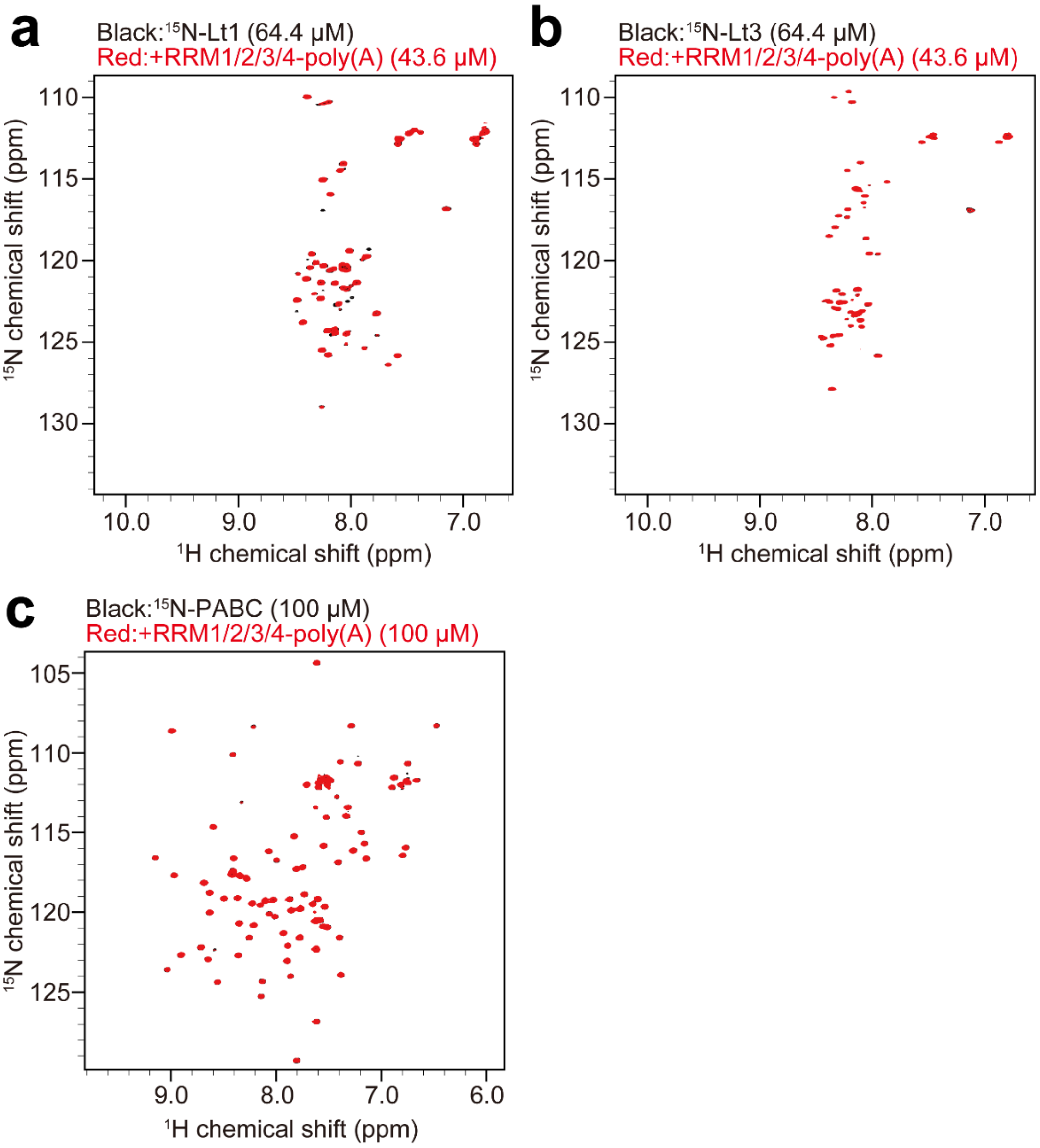

Supplementary Figure 2 ^{1}H-^{15}N HSQC spectra of Lt1, Lt3 and PABC in the presence and absence of RRM1/2/3/4-poly(A)

(a, b) ^{1}H-^{15}N HSQC spectra of ^{15}N-labelled Lt1 (a) and Lt3 (b) at 64.4 μM in the presence (red) and absence (black) of 43.6 μM RRM1/2/3/4-poly(A). (c) ^{1}H-^{15}N HSQC spectra of ^{15}N-labelled PABC at 100 μM in the presence (red) and absence (black) of 100 μM RRM1/2/3/4-poly(A).

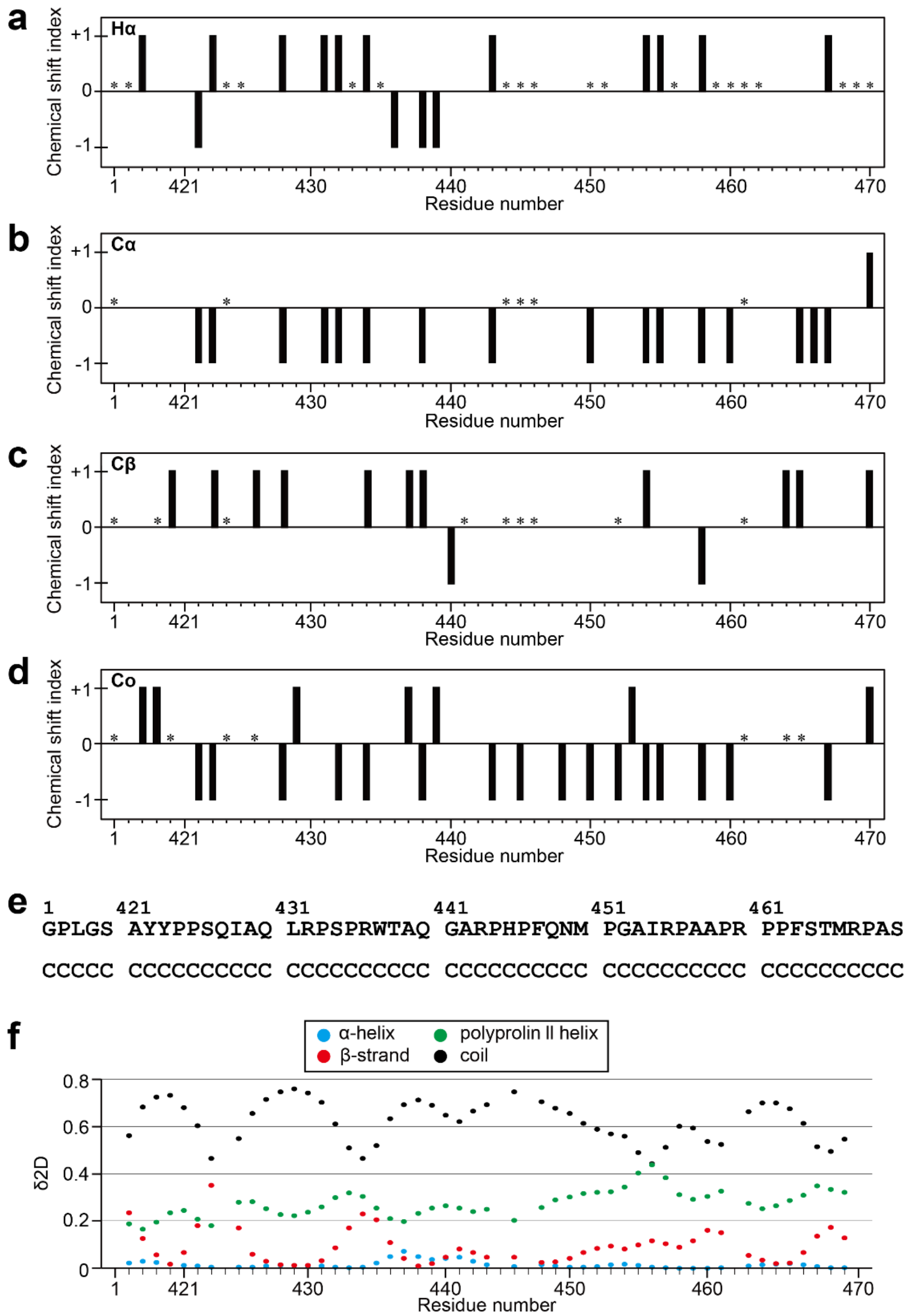

Supplementary Figure 3 Secondary structure prediction of Lt2, based on chemical shift values

Chemical shift indexes[1,2] of ^{1}Hα (a), ^{13}Cα (b), ^{13}Cβ (c) and ^{13}Co (d) were derived from chemical shift values in the chemical shift table of Lt2 (Supplementary Table 1). (e) Prediction by CSI3.0 [3]. (f) Prediction by δ2D [4]. * indicates residues without assignments. The residue numbers 1-5 are for the residues remaining upon digestion of the N-terminal tag protein.

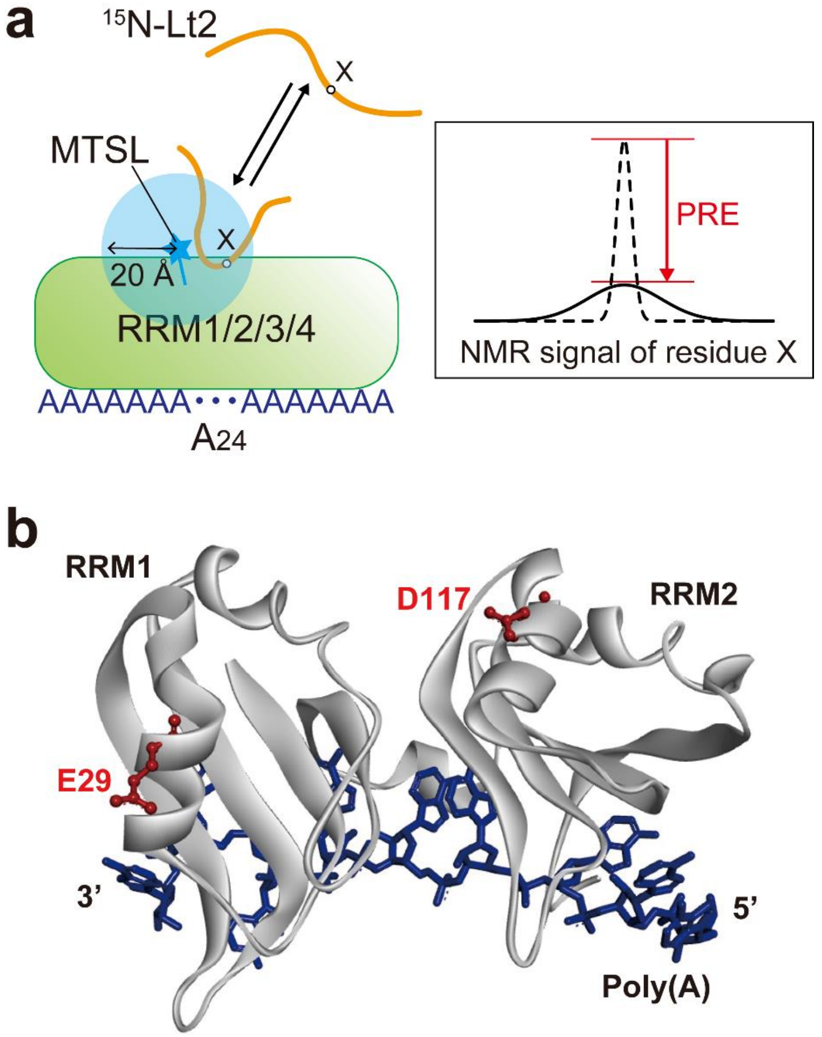

Supplementary Figure 4 Schematic representation of the paramagnetic relaxation enhancement (PRE) experiment and positions of a spin label on RRM1 or RRM2

(a) Schematic of the PRE experiment. When some residues of ^{15}N-Lt2 (residue X, depicted as an open circle) approach a spin label (MTSL covalently attached to E29C of RRM1 or to D117C of RRM2) on RRM1/2/3/4-A_{24}, within approximately 20 Å, the NMR signal of the residue X of ^{15}N-Lt2 is broadened by the PRE effect, resulting in the signal intensity reduction. (b) The positions of a spin label on RRM1 or RRM2. A spin label (MTSL) was covalently attached to a Cys residue mutated from E29 or D117 of Cys-less RRM1/2/3/4. E29 and D117 are shown in red on the crystal structure of RRM1/2 in complex with poly(A) (PDB 1CVJ[5]). It should be noted that Cys-less RRM1/2/3/4 (E29C-MTSL) and Cys-less RRM1/2/3/4 (D117C-MTSL) were separately prepared and used for the individual PRE experiment, in order to reveal which of RRM1 or RRM2 binds to Lt2.

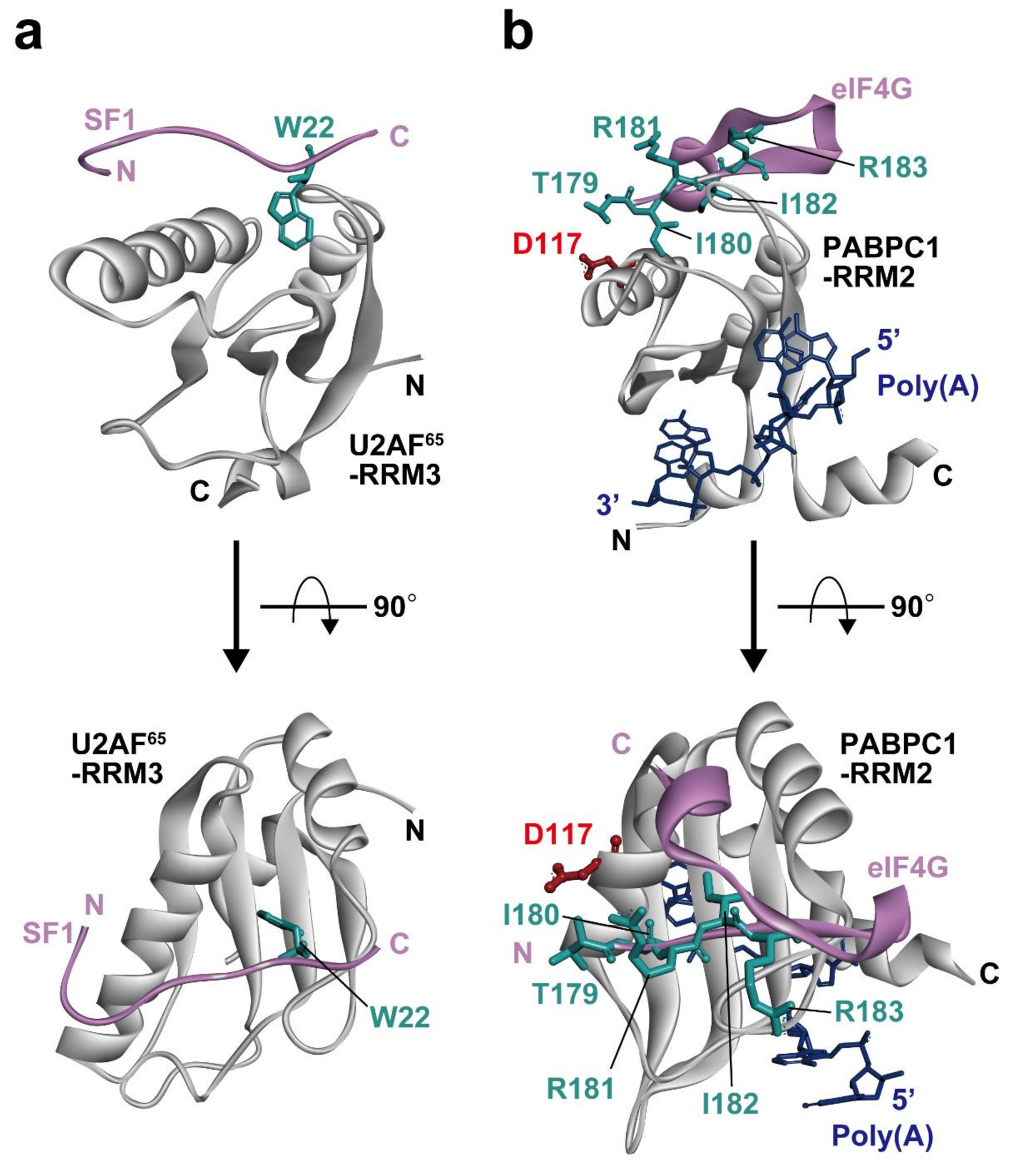

Supplementary Figure 5 A description of the binding site of the Trp-containing peptide on RRM from other protein and that of the eIF4G on RRM2 of PABPC1

(a) Structure of SF1 peptide in complex with U2AF^{65}-RRM3 (PDB 1O0P [6]). The residues 13–25 of SF1 and U2AF^{65}-RRM3 are drawn as magenta and gray ribbon representation, respectively. W22 of SF1 peptide is drawn as cyan stick representation. (b) Structure of eIF4G peptide in complex with PABPC1-RRM1/2 in the presence of A_9 (PDB 4F02[7]). The residues 179-198 of eIF4G are drawn as a magenta ribbon with cyan sticks for the sidechains that interact with RRM2 at the site corresponding to the W22-binding site in (a). PABPC1-RRM2 and poly(A) are drawn as a gray ribbon and blue sticks. D117 of PABPC1-RRM2 is colored red, in order to indicate the position of the spin label in the PRE experiment.

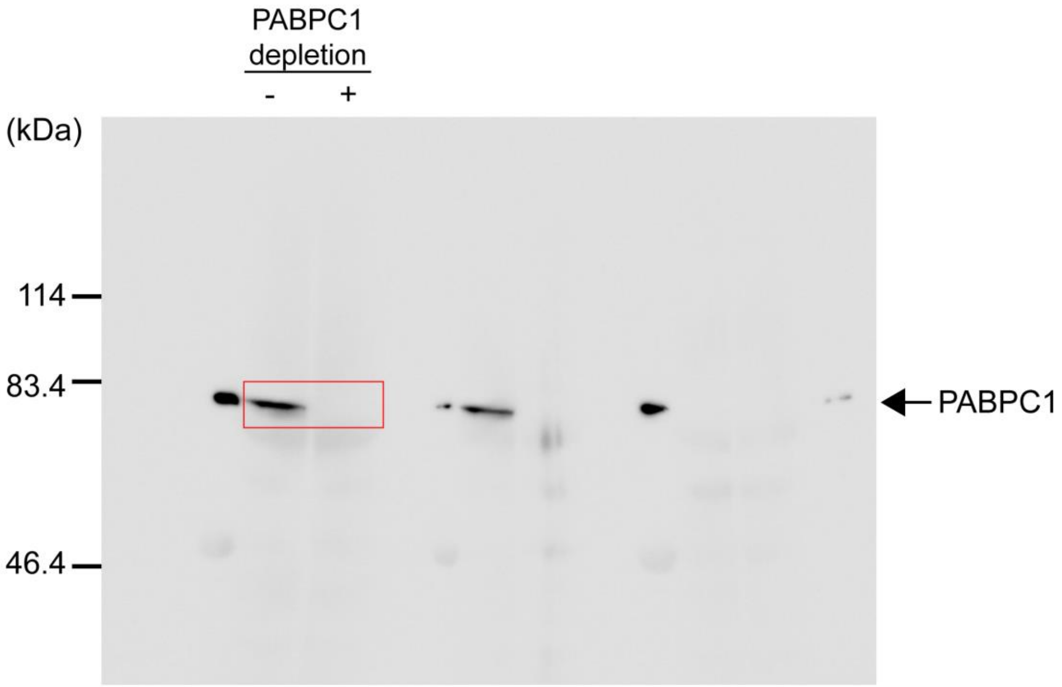

Supplementary Figure 6 The uncropped original blots of Figure 7b

Figure 7b was the image cropped in the red box.

References

1 Wishart, D. S., Sykes, B. D. & Richards, F. M. The chemical shift index: a fast and simple method for the assignment of protein secondary structure through NMR spectroscopy. *Biochemistry* **31**, 1647-1651 (1992).

2 Wishart, D. S. & Sykes, B. D. The 13C chemical-shift index: a simple method for the identification of protein secondary structure using 13C chemical-shift data. *J Biomol NMR* **4**, 171-180 (1994).

3 Hafsa, N. E., Arndt, D. & Wishart, D. S. CSI 3.0: a web server for identifying secondary and super-secondary structure in proteins using NMR chemical shifts. *Nucleic Acids Res* **43**, W370-377, doi:10.1093/nar/gkv494 (2015).

4 Camilloni, C., De Simone, A., Vranken, W. F. & Vendruscolo, M. Determination of secondary structure populations in disordered states of proteins using nuclear magnetic resonance chemical shifts. *Biochemistry* **51**, 2224-2231, doi:10.1021/bi3001825 (2012).

5 Deo, R. C., Bonanno, J. B., Sonenberg, N. & Burley, S. K. Recognition of polyadenylate RNA by the poly(A)-binding protein. *Cell* **98**, 835-845 (1999).

6 Selenko, P. *et al.* Structural basis for the molecular recognition between human splicing factors U2AF65 and SF1/mBBP. *Mol Cell* **11**, 965-976 (2003).

7 Safaee, N. *et al.* Interdomain allostery promotes assembly of the poly(A) mRNA complex with PABP and eIF4G. *Mol Cell* **48**, 375-386, doi:10.1016/j.molcel.2012.09.001 (2012).
